# Diagnosis of *Neisseria gonorrhoeae* Using Molecular Beacon

**DOI:** 10.1155/2015/597432

**Published:** 2015-01-31

**Authors:** Divya Sachdev, Achchhe Lal Patel, Subash Chandra Sonkar, Indu Kumari, Daman Saluja

**Affiliations:** Dr. B.R. Ambedkar Center for Biomedical Research, University of Delhi, Delhi 110007, India

## Abstract

*Neisseria gonorrhoeae* is an important sexually transmitted diseases (STD) causing pathogen worldwide. Due to absence of an affordable diagnostic assay, routine screening of gonococcal infection becomes impossible in developing countries where infection rates are maximum. Treatment is given on the basis of symptoms alone which leads to spread of infection. Thus, development of a rapid, sensitive, specific, and PCR based visual diagnostic assay suitable for developing countries, required for better disease management, is aimed at in present study. Endocervical swabs were collected from patients visiting gynecology department of various hospitals in Delhi. In-house PCR based assay was developed and modified to visual assay using molecular beacon for end-point detection. It was evaluated against Roche AMPLICOR NG kit and *rmp* gene. Specificity of beacon was confirmed by competition experiments. Diagnostic test was 98.21% specific and 99.59% sensitive whereas negative and positive predicted value were 99.40% and 98.78%, respectively. We also observed that twice the concentration (2X) of premix was stable at 4°C for 4 months and dry swab samples gave concordant results with that of wet swabs. These features make the test best suitable for routine diagnosis of genital infections in developing countries.

## 1. Introduction

*Neisseria* is one of the most common STD causing pathogens. WHO estimated that 106 million cases occur annually with approximately 2/3rd of these cases reported from developing countries [1]. Although easily curable by a single dose of antibiotic, large population goes undiagnosed due to absence of rapid, sensitive, specific, and cost effective diagnostic methods. Early diagnosis and treatment can also reduce the risk of the patient developing long-term complications of the disease. The available diagnostic methods like gram stain and culture are economical but have poor sensitivity especially in asymptomatic patients. Moreover, delay in availability of the results makes these methods not only unsuitable for screening but also unsuitable for treatment as a number of patients (especially adolescents and pregnant women) do not return to collect their results [2, 3]. NAAT based commercial kits are also available for diagnosis of* Neisseria* but, due to the high cost, these do not meet the requirement of mainstream laboratories in developing countries where majority of world population is afflicted. Syndromic management introduced by WHO not only lacks both sensitivity (30%–80%) and specificity (40%–80%) for the infections in women with vaginal discharge [2, 4, 5] but also misses asymptomatic patients [6, 7]. A recently introduced POC test, PATH GC check rapid test (Program for Appropriate Technology, Seattle, USA), is an immunochromatographic strip test, with moderate sensitivity ranging from 81% to 31.2% under different clinical setting as compared to Roche AMPLICOR CT/NG PCR assay and 16 s rRNA PCR assay [8]. Highly sensitive real time PCR kits recently approved for in vitro diagnosis are exceedingly expensive and out of reach of even best clinical laboratories in the developing world. Therefore, there is an urgent need for a sensitive, rapid, cost effective, and point-of-care test in developing countries for routine detection of* Neisseria* so as to prevent the widespread infection and development of reproductive sequelae.

In the present study, we have modified the previously developed PCR assay for the detection of* Neisseria* [9] so as to make it more specific, sensitive, and easy to use. By using molecular beacon for end-point detection, the assay eliminates agarose gel electrophoresis to visualize the amplicon, increases specificity of assay by hybridizing to the internal sequence of the amplicon, and minimizes the risk of cross contamination and carry-over contamination to other clinical samples [10]. Further, we have stabilized the PCR mix at 4°C for up to 4 months as well as standardizing the use of dry swabs for collection of samples in empty vials, which can easily be transported at room temperature (up to 30°C), making it a method of choice in peripheral laboratories with minimum resources [11]. In brief, the molecular beacon based PCR assay described here meets the essential requirements for use in resource limited settings.

## 2. Materials and Methods

### 2.1. Patient Population

Endocervical swab samples from nonpregnant women (18 years–55 years of age with 90% of patients in the age group of 18–31 years) seeking diagnosis and treatment of vaginal discharge syndrome (VDS) or PID were collected during 2008-2009 and 2011-2012 from the outpatient departments of gynaecology from various hospitals, Delhi, as per the guidelines of Indian Council Of Medical Research, India, and adopted by Institutional Ethical Committee of Dr. B.R. Ambedkar Centre for Biomedical Research, University of Delhi (number F50-2/Eth.Com/ACBR/11/2107). Patients on antibiotic treatment were excluded from the study. Written informed consent was taken from all patients enrolled in the study.

### 2.2. Specimen Collection and Processing

After thorough speculum examination of vulva for warts, ectopy, and discharge by the attending clinician, two endocervical swabs were collected from each patient. Our study was divided into two groups. For group 1 (*n* = 412), the first swab was placed in a vial containing AMPLICOR specimen transport medium and the second swab was placed in 1 mL transport medium (phosphate buffer saline with 1 mM EDTA) [12, 13]. For group 2 (*n* = 133), the first swab was placed in transport medium and the second swab was placed in the empty vial (dry swab). All the wet swab specimens were transported on the same day to microbiology laboratory on ice while the dry swabs were transported at room temperature (25°C–30°C) and were either tested on the same day or stored at subzero temperature till further use. Dry swabs were immersed in PBS (1 mL) for 10 min before processing. All samples were processed by enzymatic lysis method as described previously [13, 14].

### 2.3. Primers and Probes Used

The primers used in our previous studies designed against the* orf1* gene sequences [9] were modified to improve specificity and sensitivity of the assay. The modified primers (orf1F 5′-GATCCAACTATTCCCGATTGC-3′ and orf1R 5′-GCAAAGTTATACAGCTTCGCCTGA-3′) for* orf1* gene of* N. gonorrhoeae* produced an amplicon of size 269 bp. Molecular beacon, 5′-Fluorophore -CCATGCG TGACTGCCAACAAGAAAAAAGCCATCC CGCATGG-Quencher-3′ (Eurogenetic, Belgium; Tm = 69.7°C) complementary to region within amplicons of* orf1* gene, was designed as described by Tyagi and Kramer [15] with melting temperature of beacon 5–7°C higher than annealing temperature of primers. Quencher fluorophore pair was chosen such that emission spectrum of fluorophore overlapped with absorption spectrum of quencher. Two different fluorophore-quencher pairs, Cy3-Dabcyl and FAM-BHQ2, were used.

### 2.4. PCR Amplification

Supernatant (5 *μ*L) of processed sample or crude lysate was used for PCR [13, 14]. The PCR mix contained 50 mM of KCl, 10 mM of Tris-Cl (pH 8.3), 2.0 mM of MgCl_2_, 200 *μ*M of each of the four dNTPs (New England Biolabs Inc.), 10 pmoles of each of forward and reverse primers, and 1 U of Taq DNA polymerase (Bangalore Genei India Pvt. Ltd., India). Negative (no template) and positive controls (*orf1* gene cloned in TA vector) were included in each PCR run. Amplification of* orf1* gene was performed in thermal cycler (Bio-Rad) for 35 cycles with the following parameters: 95°C for 5 min for initial denaturation, cycling of 95°C for 30 sec, 60°C for 30 sec, 72°C for 30 sec, and final extension at 72°C for 10 min. The amplicons were analyzed by agarose gel (1%) electrophoresis {Figure S1 (see Supplementary Material available online at http://dx.doi.org/10.1155/2015/597432)}.

The amplicons from positive samples (10% of the total) were eluted from agarose gel using DNA isolation kit (Geneaid, USA) according to the manufacturer's instructions and sequenced using PCR primers. DNA sequence of the amplified product was compared to the known* orf1* nucleotide sequences (AE004969.1, NGO0365) in the GenBank databases using BLAST program to determine the percent identity.

### 2.5. Roche AMPLICOR MWP* Neisseria gonorrhoeae* Detection Assay

412 endocervical specimens (group 1) were tested by Roche AMPLICOR Multiwell plate NG detection kit (Roche Diagnostic Systems) according to the manufacturer's instructions.

### 2.6. Use of Molecular Beacons

Asymmetric PCR was standardised using 40 pmoles of sense primer and 20 pmoles of antisense primer amplifying more copies of strand complementary to probe [16]. Complementary strands generally anneal at higher temperature than molecular beacon hybridising to its target during ramping. Asymmetric PCR leaves higher amount of target strand for beacon to hybridize. This reduces the competition of beacon with its target and thus helps in obtaining better signal and minimum noise [16]. The amplified product and beacon mix was again heated to 95°C for 5 minutes and was slowly cooled to 20°C at ramp rate of 0.1°C/second. The temperature cycle added to PCR cycling conditions leads to maximum binding of beacon to its target sequence. Unbound probe hybridises back to hair pin shape as temperature slowly cools below its melting temperature, thus reducing the noise. The products were checked by (i) fluorescent ELISA reader, (ii) dark reader, and (iii) agarose gel electrophoresis. To visualize the amplicons using ELISA reader, the reaction contents (50 *μ*L) were transferred into 96-well plate. To each well, 150 *μ*L of 20 mM Tris/Cl (pH 7.4) was added and the fluorescence was measured using appropriate excitation/emission wavelength pair [17] using a spectrofluorometer (M200 infinite, Tecan Group Ltd.) at 37°C. Clinical samples (*n* = 412) were tested using Fam-BHQ2 pair and randomly selected clinical samples (*n* = 80) were tested using Cy3-Dabcyl pair. The excitation wavelength of Fam is 491 nm and emission wavelength is 521 nm, and excitation wavelength for Cy3 is 554 nm and emission wavelength is 568 nm. In method (ii), the tubes were placed in the slot of in-house dark reader manufactured by DSS Tech Pvt. Ltd. for detection of fluorescence by visual inspection.

### 2.7. Evaluation of Sensitivity and Specificity of Molecular Beacon

Different concentrations of molecular beacon (0.2 pmoles to 1 pmole) were used to standardise the concentration with minimum noise and maximum signal. Further, to evaluate the sensitivity of molecular beacon, serial dilutions from 100 ng to 10 fg of purified* Neisseria* genomic DNA were used. We have also used nonspecific unlabeled probe in excess (specific : nonspecific ratio ranging from 1 : 1 to 1 : 50) along with specific probe in PCR reactions. All assays were repeated at least five times.

### 2.8. Stabilisation of PCR Reaction Mixture at 4°C

Master mix containing all the reagents other than template in twice the concentration was stored at 4°C for different time intervals. An aliquot of master mix was checked at zero time for the activity of the enzyme. Aliquots of master mix were checked at regular intervals till seven months for the PCR assay.

### 2.9. Statistical Analysis

All of the statistical analysis was performed using GraphPad prism 5 software (GraphPad Software, Inc.).

## 3. Results

### 3.1. Clinical Performance of the In-House PCR Using Modified Primers

Primers published earlier were slightly modified for carrying out detection using molecular beacon. Efficacy of in-house PCR for* N. gonorrhoeae* was evaluated against Roche AMPLICOR MWP kit and* rmp* gene [18]. All samples were tested using modified primers, Roche AMPLICOR MWP kit, and published primers for rmp gene using swab samples collected from 412 symptomatic women patients with the median age of 24 years. A sample testing positive with two or more methods was scored positive whereas sample testing negative with two or more methods was considered to be negative to calculate the performance characteristics of the test. The modified* orf1* primers were found to be 97.02% sensitive and 99.18% specific (Table 2).

### 3.2. Clinical Evaluation of Molecular Beacon

**Figure 1:**
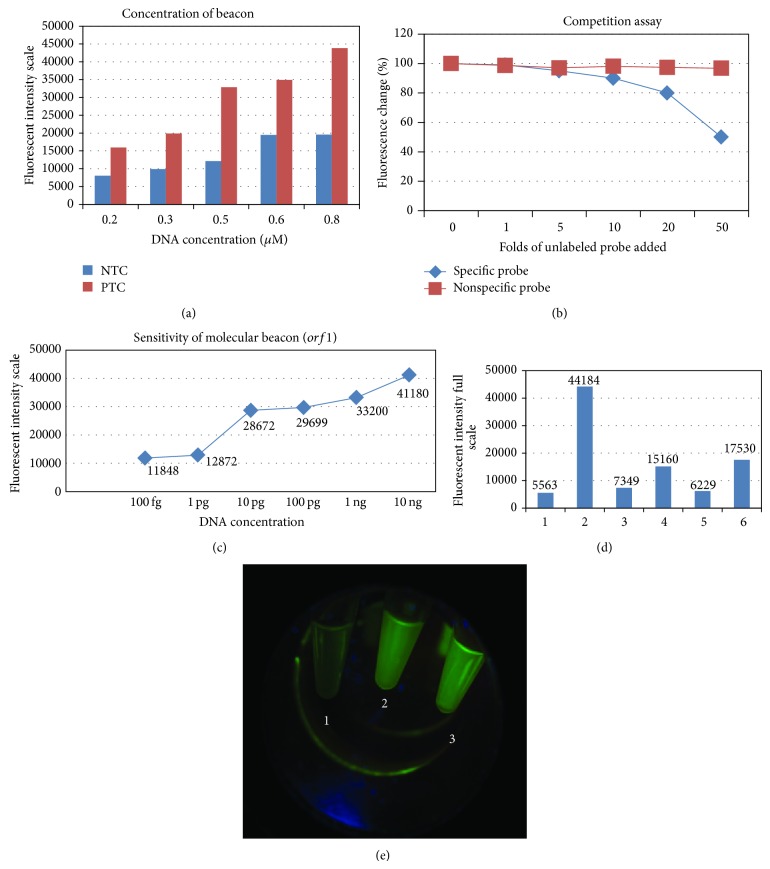
Standardisation of use of moecular beacon for detection of* N. gonorrhoeae*. (a) Standardization of concentration of beacon. (b) Specificity of molecular beacons using unlabeled specific and nonspecific probe (1 : 0, 1 : 1, 1 : 5, 1 : 10, 1 : 20, and 1 : 50). (c) Sensitivity of molecular beacon using purified neisserial genomic DNA (100 fg to 10 ng). (d) Detection of clinical samples in ELISA reader. The amplified PCR products were transferred into wells of a 96-well plate containing 150 *μ*L of 10 mM Tris/Cl (pH 8.0) and the fluorescence was measured using 492 nm excitation and 521 nm emission in ELISA reader. (e) Direct visualization of PCR tube under dark reader. Tube 1: NTC, tube 2: PTC, and tube 3: clinical sample (positive).

Optimal concentration of beacon was standardized at 0.8 pmoles which gave maximum signal and minimum noise (Figure 1(a)) and beacon was found to be sensitive till 100 fg of gDNA (Figure 1(c)). We observed that fluorescence decreased to 60% on addition of 40-fold of unlabeled specific probe whereas it remained unchanged on addition of unlabeled nonspecific probe (Figure 1(b)). A total of 412 clinical samples were checked for presence of* Neisseria* using Fam labelled molecular beacon detected using ELISA reader as well as dark reader (Figures 1(d) and 1(e)). The second pair of fluorophore-quencher (Cy3-Dabcyl) was also used for detection of randomly selected clinical samples (*n* = 80) which produced similar results (Figure S2) suggesting that molecular beacon worked effectively independent of the fluorophore-quencher pair. Out of 412 samples, 154 tested positive for* Neisseria gonorrhoeae* using the above four detection methods. There were three samples which were positive by agarose gel electrophoresis, molecular beacon, and* rmp* gene but tested negative by Roche AMPLICOR kit and were considered as true positive based on composite reference standard method. There were four samples which tested negative by in-house PCR but were positive by molecular beacon method as well as by Roche AMPLICOR kit and rmp gene PCR. Two samples were false positive by in-house PCR as they tested negative by molecular beacon and by another two detection methods used. Thus, use of molecular beacon enhanced the sensitivity of our test. Only one sample falsely tested positive using molecular beacon (Table 1). Based on composite reference standard method, use of molecular beacon enhanced the sensitivity of our test to 98.21% and specificity to 99.21%. Our test showed accuracy of 99.01%. We conclude that use of molecular beacons for detection of amplicons not only reduces time of detection but also increases the sensitivity (due to fluorescence) and specificity (due to hybridization of the beacon) of the diagnostic tests with practically no false positives or false negative results (Table 2).

### 3.3. Use of Dry Swabs for Clinical Evaluation

Since it is easier to transport dry swab samples, we checked the performance of in-house PCR using dry swabs (*n* = 133) and found similar sensitivities of PCR assay to those of gDNA isolated from wet swabs. Out of 133 samples, 33 were positive when total DNA was extracted from dry or wet swabs while 96 were negative for* Neisseria gonorrhoeae* infection. Discordance in PCR results was observed for 4 samples using dry and wet swabs. All samples were also analysed using housekeeping gene,* rmp* (as shown in Table 3), which helped in resolving discrepant results. We found good concordance of results with dry and wet swabs; thus use of dry swabs is recommended as a preferred method for sample collection.

### 3.4. Standardisation of Reagents at 4°C

**Figure 2:**
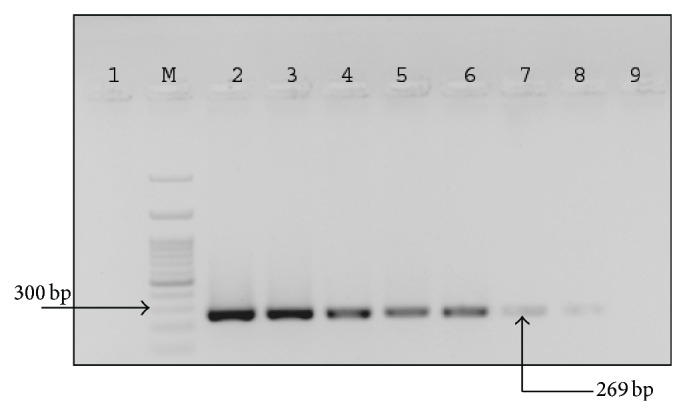
Agarose gel showing the stability of PCR master mix at 4°C. Amplification of* orf1* gene was performed with PCR master mix stored at 4°C for 0, 1, 2, 3, 4, 5, 6, and 7 months (Lanes 2–9, resp.). Lane 1 is no template control. Lane M is 100 bp DNA ladder. Master mix is found to be stable and functional for four months.

Further, to qualify an assay as user friendly and affordable diagnostics, premix of the reagents (buffer, dNTPs, primers, probe, and Taq DNA polymerase) was made at twice the concentration and kept at 4°C for up to 7 months. PCR amplification was carried out at regular intervals and we found that premix was stable up to 4 months, making it an easy assay to be carried out in peripheral laboratories where deep freezers are not available (Figure 2).

## 4. Discussion

Diagnosis by a clinician can sometimes be made on the basis of signs and symptoms of the disease but accurate diagnosis requires a specific diagnostic test, often requiring access to a clinical laboratory. A reasonably good and accurate diagnostic test is therefore of paramount importance in reducing the burden of infectious diseases. For the past two decades, nucleic acid based PCR assays have been profusely published as diagnostic methods [12–14, 19, 20]. PCR methods are highly specific and sensitive and less time consuming than culture method. In spite of this, PCR based diagnostic assays have failed to penetrate the market in developing countries. To be useful, diagnostic methods must be accurate, simple, and affordable for the population for which they are intended. Due to the high cost of PCR based diagnostic methods and lack of infrastructure and expertise available in resource-limited setups, developing countries are still using assays which have low sensitivity and specificity. A conventional PCR based assay can result in false positives and experimental variability. Although automatic systems, which combine nucleic acid extraction with high throughput PCR amplification, reduce problems associated with manual sample processing and visualization, they are highly expensive (more than $140,000) and out of reach of even best clinical laboratories in the developing world. Most of these tests use real time techniques which not only are expensive but also require technical expertise and are time consuming. The present study was focussed on designing of an easy visualization based method for the detection of* Neisseria gonorrhoeae* using molecular beacon. Not only is our in-house PCR test as sensitive as that of CT/NG Roche AMPLICOR Multiwell plate kit but use of molecular beacon has made detection far easier. In our test, DNA amplification was performed in routine thermal cycler followed by end-point detection using molecular beacon on dark reader or ELISA reader. The amplicons can be detected by hybridization to fluorescent tagged molecular beacon and tubes can be visualised directly under an indigenous dark reader (costing around $1000). Since, in most of the diagnostic laboratories, presence or absence of pathogen is required for deciding treatment, the end-point detection by dark reader is adequate. Visualising the PCR products directly in the PCR tube decreases the time of detection and also minimizes the chances of cross contamination as well as carry-over contamination which is highly beneficial to the user.

In many developing countries, protocols for waste disposal and biosafety either are not developed or are highly rudimentary. Containment of biohazardous material such as ethidium bromide is almost nonexistent [20]. The diagnostic assay described in this study eliminates the running of agarose gel which is time consuming and cumbersome and uses carcinogenic chemicals like EtBr. Collection of dry swabs for clinical samples at ambient temperature (25°C–30°C) makes it easy to maintain the temperature during transportation of samples from field to nearby laboratories which further helps in reduction of cost spent for sample collection as well as transportation. Similarly, use of PCR reaction premix (containing PCR buffer, primers, and molecular beacon as well as Taq polymerase) reduces the need for training end user of micropipetting. The use of premix will also eliminate the chance of contamination and improves consistency and repeatability by even untrained worker. The premix is also stable for at least four months at 4°C. Since it is easier to maintain 4°C in fridges (also during electricity failure one can use cool packs, a situation quite common in resource-poor settings and in rural areas), it is of great benefit for peripheral laboratories with minimal resources. Use of molecular beacon and dry swabs for sample collection and use of dark reader to visualise results are expected to decrease the cost of test tremendously. The patients using Roche AMPLICOR kit based test for diagnosis of* Neisseria* are charged Rs 1500–2000 in different laboratories in India, whereas our laboratory cost of our test is Rs 60 only. This amount falls in affordable range of large percentage of population of India and other developing countries, thus helping in better diagnosis and efficient treatment and disease management. In a nutshell, the molecular beacon based PCR assay developed in the present study addresses to a large extent the need of a simple, cost effective, easy to use, highly sensitive, and specific nucleic acid amplification assay, a step forward towards developing point-of-care diagnostics for low resource settings [21, 22]. To the best of our knowledge, this is the first report of use of molecular beacons for diagnosis of* Neisseria gonorrhoeae*. Future work is in progress to address the major challenge to automate the DNA extraction and PCR assay at an affordable price. Local production of such diagnostic kits will further reduce the cost. Faster, simpler, and cost effective diagnostics will make disease control efforts more effective especially in places where patients have difficulty in reaching health care.

## Supplementary Material

Figure S1: Agarose gel (1.5%) detecting the infection load in clinical samples using in house PCR.
Figure S2: Comparative analysis of use of different fluorophone - quencher pairs for molecular beacon for detection of *N. gonorrhoeae*.

## Figures and Tables

**Table 1:** Comparison of use of molecular beacon as detection method with gel electrophoresis, Roche AMPLICOR MWP kit, and PCR with a housekeeping gene: *rmp*.

Number of samples	PCR	Beacon	Roche	*rmp *	Conclusion
154	+	+	+	+	Positive
4	−	+	+	+	Positive
2	−	−	+	−	Negative
1	−	+	−	−	Negative
3	+	+	−	+	Positive
2	+	−	−	−	Negative
4	+	+	+	−	Positive
2	+	−	+	+	Positive
1	−	−	+	+	Positive
2	−	−	−	+	Negative
237	−	−	−	−	Negative

**Table 2:** Comparison of sensitivity, specificity, positive predictive value (PPV), negative predictive value (NPV), likelihood ratios (LR), and accuracy of different methods calculated with 95% confidence intervals (CI).

Result of samples (*n* = 412)	PCR		Beacon		Roche AMPLICOR MWP kit		Rmp	
	Positive	Negative	Positive	Negative	Positive	Negative	Positive	Negative
NG positive	163	5	165	3	165	3	164	4
NG negative	2	242	1	243	2	242	2	242
Sensitivity (95% CI)	97.02	93.19–99.02	98.21	94.86–99.61	98.21	94.86–99.61	97.62	94.01–99.33
Specificity (95% CI)	99.18	97.06–99.88	99.59	97.73–99.93	99.78	97.06–99.88	99.81	97.06–99.88
PPV (95% CI)	98.79	95.68–99.82	99.40	96.68–99.90	98.80	95.73–99.80	98.8	95.71–99.82
NPV (95% CI)	97.98	95.34–99.33	98.78	96.47–99.73	98.78	96.46–99.73	98.37	95.89–99.55
LR (+)	118.37	29.76–470.74	239.64	33.89–1694.66	119.82	30.13–476.46	119	29.95–473.60
LR (−)	0.03	0.01–0.07	0.02	0.01–0.06	0.02	0.01–0.06	0.02	0.01–0.06
% accuracy	98.3		99		98.7		98.5	

**Table 3:** Comparison of in-house PCR using dry swabs with wet swabs as mode of sample collection for *N. gonorrhoeae*.

Clinical samples (*n* = 133)	PCR with orf1 primers		rmp	Conclusion
	Wet swabs	Dry swabs		
33	+	+	+	Positive
96	−	−	−	Negative
2	−	+	+	Positive
1	+	−	−	Negative
1	+	−	+	Positive
