# Supplementary material for: Diagnosis of Neisseria gonorrhoeae Using Molecular Beacon
Source: Biomed Res Int. 2015 Jan 31;2015:597432. doi: 10.1155/2015/597432 (PMC4329845; doi:10.1155/2015/597432)
Supplement: Supplementary file 1 — Figure S1: Agarose gel (1.5%) detecting the infection load in clinical samples using in house PCR. Figure S2: Comparative analysis of use of different fluorophone - quencher pairs for molecular beacon for detection of N. gonorrhoeae. [file 597432.f1.pdf]

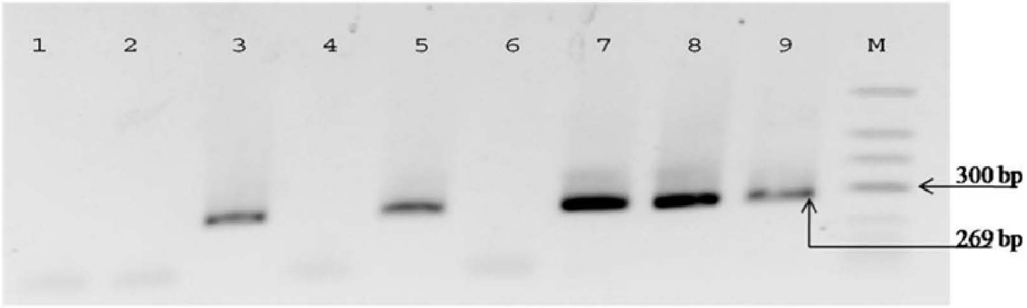

Figure S1: Agarose gel (1.5%) detecting the infection load in clinical samples using in house PCR: Amplicon of 269 bp (indicated by arrow) shows successful amplification of orf1 of *N. gonorrhoeae* Lane 1 is No template control, lane 2-8 are clinical isolates and Lane 9 is positive template control.

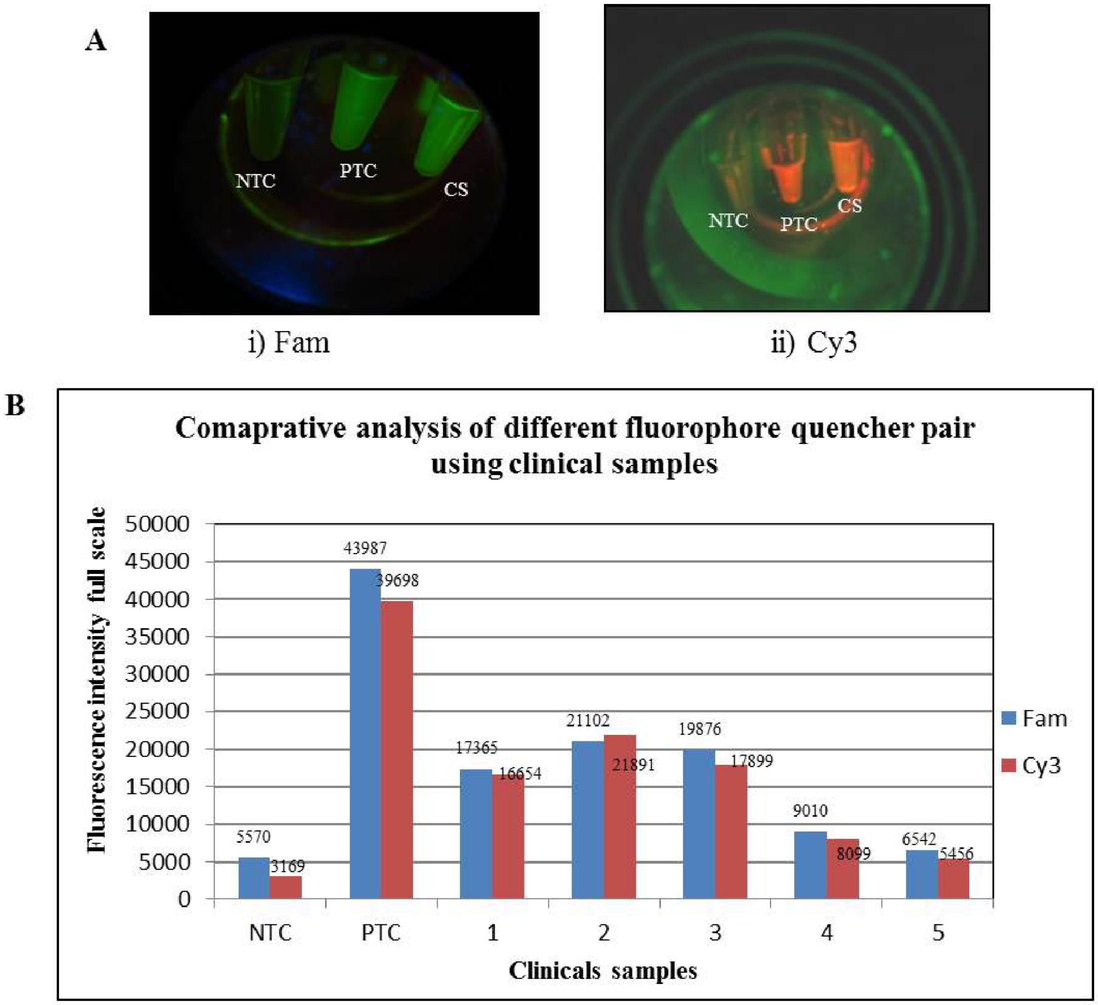

Figure S2: Comparative analysis of use of different fluorophore - quencher pairs for molecular beacon for detection of *N. gonorrhoeae*.

A) Direct visualization of PCR tube under dark different reader. NTC = No template control, PTC = Positive template control, CS = clinical sample.

B) Detection of different clinical samples in ELISA reader using different fluorophores.
